# Evaluation of usability, feasibility and acceptance of the digital training diary *Trainingslog* for individuals with axSpA: a mixed-method study

**DOI:** 10.1186/s41927-025-00463-5

**Published:** 2025-02-18

**Authors:** Neva Pfyl, Lea Ettlin, Karin Niedermann, Anne-Kathrin Rausch

**Affiliations:** 1 ZHAW Zurich University of Applied Sciences, Institut of Physiotherapy, Katharina-Sulzer-Platz 9, Winterthur, 8401 Switzerland; 2 Swiss Ankylosing Spondylitis Association, Leutschenbachstrasse 45, Zurich, 8050 Switzerland; 3 ZHAW Zurich University of Applied Sciences, Institut of Public Health, Katharina-Sulzer-Platz 9, Winterthur, 8401 Switzerland

**Keywords:** Axial spondyloarthritis, Physical activity, Training diary

## Abstract

**Background:**

Physical activity (PA), including regular exercise, is essential for the successful management of axial spondyloarthritis (axSpA). To promote a physically active lifestyle, a digital training diary (*Trainingslog*) was developed in an user-centered approach by the Swiss Ankylosing Spondylitis Association (SVMB). A training diary promotes PA through feedback, goal setting and self-monitoring, which can also be used for PA counselling by physiotherapists (PT). Usability, feasibility and acceptance are essential for the successful implementation of a mobile Health Intervention such as the *Trainingslog*. The study objective is to evaluate the usability, feasibility and acceptance of the *Trainingslog* for individuals with axSpA and PTs.

**Methods:**

A mixed-methods design was performed among potential end-users of the *Trainingslog*. Quantitative data was collected by use of questionnaires (System Usability Scale (SUS, 0-100 scale), user version of the Mobile App Rating Scale (uMARS, 5 point scale)) and number of training entries. Subsequently, qualitative data was gathered through semi-structured online focus groups or individual interviews.

**Results:**

11 PTs (9 women, mean age 52.5 [SD 15.6]) and 10 individuals with axSpA (6 women, mean age 48 [SD 13.4]) participated. The quantitative data showed mean SUS scores for usability of 82.5 [SD 21.76] for PTs and 77.0 [SD 9.34] for individuals with axSpA. The mean uMARS sector B scores for feasibility were 4.2 [SD 0.49] for PTs and 4.1 [SD 0.38] for individuals with axSpA. Acceptance, as indicated by the uMARS results (mean score > 3 in Sectors E and F for both groups), was given. But there was a lower-than-expected agreement in the training entries, with 59.86% of entries matching between the *Trainingslog* and the paper diary. The qualitative analysis unveiled that while usability and feasibility were good, acceptance was lower, primarily due to the use of a web-based link instead of an app version.

**Conclusion:**

The *Trainingslog* showed a good usability and feasibility, while the acceptance was lower than expected. Acceptance could be increased by offering the *Trainingslog* as an app-based version, along with implementing additional recommendations for enhancement. Consequently, the *Trainingslog* has the potential to be applied in PA counselling by PTs or as a self-monitoring tool for individuals with axSpA.

**Supplementary Information:**

The online version contains supplementary material available at 10.1186/s41927-025-00463-5.

## Background

Axial spondyloarthritis (axSpA) is a chronic inflammatory rheumatic disease with multiple clinical manifestations that primarily affect the spine, the sacroiliac joints and the axial skeleton [1]. Individuals with axSpA typically suffer from back pain and spinal mobility limitation, which can lead to a decrease in quality of life and an inability to work. Furthermore, inflammatory rheumatic diseases are also associated with an increased risk of cardiovascular diseases [2, 3]. Evidence showed that regular exercise has a positive impact on disease activity, quality of life and symptoms in individuals with axSpA [4–6]. Therefore, in addition to pharmacological therapy, regular exercise is an essential component of the disease management [4, 7]. Physical activity recommendations for adults state a minimum of 150–300 min of moderate or 75–150 min of intensive aerobic exercises per week plus muscle strengthening involving all major muscle groups twice a week [8]. Furthermore, the American College of Sports Medicine (ACSM) recommends the performance of balance or agility training on at least two days of the week [9]. These recommendations were proven to be safe and feasible for individuals with axSpA [7]. However, Liu et al. reported that approximately one-third to one-half of individuals with axSpA do not comply with the public health physical activity recommendations [10]. Therefore, it is important to encourage individuals with axSpA to exercise for disease management and secondary prevention. In Switzerland, The Swiss patient organization for individuals with axSpA (Schweizerische Vereinigung Morbus Bechterew, SVMB) provides a physical activity promotion concept, called BeFit, involving exercise, assessments and counselling in group and individual settings [11, 12].

### Digital training diary

Physical activity can be supported by self-regulatory measures such as self-monitoring, feedback and goal setting [13]. Individuals with axSpA display greater adherence to exercise when they are regularly monitored [14]. Accordingly, a training diary could provide access to self-regulated training and support monitoring. Nevertheless, a previous evaluation of the SVMB offering BeFIt showed that a suitable digital training diary covering the four training dimensions of aerobic, strength, flexibility and neuromotoric exercises would be favourable, as the used paper-based training diary proved to be impractical [11]. As a result, a digital web-based training diary called *Trainingslog* covering all four training dimensions of physical activity was developed. It provides information, feedback and links to a library of exercise-programs [15].

The *Trainingslog* was not only designed for individuals with axSpA but also for physiotherapists (PTs) providing exercise counselling. The collected data from the *Trainingslog* are stored in a database and can be used for future research [11]. According to the EULAR recommendations for the development, evaluation and implementation of mobile health applications aiding self-management in people living with rheumatic and musculoskeletal diseases (RMDs), individuals with RMDs and relevant healthcare providers should be involved in all stages of the development and validation of self-management apps [16]. Therefore, the *Trainingslog* has been developed using a user-centred approach, in which the opinions of users are taken into account at every stage of the development process. User testing leads to improved usability and user acceptance [17].

In a first step, a mock-up version of the *Trainingslog* was developed by a team of experts for research methodology, technology development, physical activity, and an individual with axSpA (patient involvement) and subsequently tested by two PTs and two individuals with axSpA. Based on the evaluation of the mock-up version, the first version of the *Trainingslog* was developed and is subject of the current study.

For the acceptance and use of a digital health intervention the evaluation of usability is a key factor [18]. In order to increase the quality of the mobile health intervention, as well as the adherence of the end-users, knowledge of usability, feasibility and acceptance of the *Trainingslog* is essential. For this study, the definition of usability and acceptance by Bevan et al. was used, which operationalizes usability in terms of user performance, satisfaction and measures acceptability as part of usability in terms of product use [19]. According to Bevan et al., acceptance means ‘whether the product is used in the real world’, which is why we recorded how many training entries were in fact made [19]. Feasibility is described by Proctor et al. as the extent to which an innovation can be successfully used or implemented in a particular institution or setting [20].

The aim of this study was to evaluate the usability, feasibility and acceptance of the *Trainingslog* for individuals with axSpA and PTs. An additional objective was to formulate recommendations for further development of the *Trainingslog*.

## Methods

Based on the importance of end-user feedback, the study has an explanatory sequential mixed-methods design and was performed among the potential end-users of the *Trainingslog*. First, the quantitative data was collected by questionnaires. “The System Usability Scale (SUS) [21] and Sections A (engagement), B (functionality), and C (aesthetics) of the User Version of the Mobile Application Rating Scale (uMARS) [22] were employed as quantitative measurements for assessing usability. Additionally, uMARS [22] Section B was utilized to evaluate feasibility. To assess acceptance, Sections E (app subjective quality) and F (app-specific questions) of the uMARS [22], along with the number of reported trainings, were used. Second, in order to complement the quantitative data, qualitative data was recorded through semi-structured focus group interviews and individual interviews conducted online.

Ethical approval was obtained from the Zurich Ethics Committee (BASEC-Nr. 2022-00578).

### Participants/recruitment

The study population were the end-users of the *Trainingslog*. The study sample consisted of two groups composed of group leaders of SVMB therapy-groups (PTs) and individuals with axSpA in the German-speaking area of Switzerland. Based on the sample size recommendation for usability studies of Macfield, a sample size of 10 +/-2 people per group was aimed for [23]. For both potential user-groups, PTs and individuals with axSpA, the inclusion criteria were age over 18 years, a good knowledge of German and possession and use of a smartphone. People with a neurophysiological disorder that leads to severe psychiatric disorders, cognitive impairment or inability to communicate were excluded from the study.

The recruitment of the study sample took place between July 2022 and December 2022. Therefore, a recruitment e-mail was sent to all SVMB members and group leaders by the SVMB. In addition, potential subjects were recruited directly on-site during the regular BeFit exercise sessions or were contacted personally via e-mail.

### Data collection

After providing written informed consent, written instructions and questionnaires were sent to the study participants. In the case of questions and uncertainties, participants received support by e-mail or telephone during the entire test period.

The group of individuals with axSpA was instructed to test the *Trainingslog* for three weeks and to use it every day in their usual routine. Before these test weeks, the participants completed a questionnaire on their proficiency related to technology (Mobile Device Proficiency Questionnaire (MDPQ)). During these three weeks, they were reminded weekly to fill in the paper-training diary. After the test phase, the participants filled in two more questionnaires (User Version of the Mobile Application Rating Scale (uMARS) and System Usability Scale (SUS)) and returned them by mail.

The PTs were asked to test the *Trainingslog* for at least 20 min, as required by uMARS [22]. After testing, they completed the questionnaires (MDPQ, uMARS and SUS) and returned the documents by mail.

After the test phase, all participants were invited to take part in an online focus group.

### MDPQ-16

The MDPQ-16 questionnaire is used to classify technology proficiency [24]. The participants were categorized in high (MDPQ-16 ≥ 48 points) and low technology proficiency groups (MDPQ-16 < 48 points) based on the score achieved. The questionnaire was translated into German using the TRAPD method and reviewed by a professional translator [25].

### uMARS

The Mobile Application Rating Scale (MARS) questionnaire measures the quality of health apps in the four objective dimensions (Section A: engagement, Section B: functionality, Section C: aesthetics, Section D: information) and two subjective dimensions (Section E: app subjective quality and Section F: app-specific items). All questions are rated on a five-point scale from 1 “Poor” to 5 “Excellent” [26]. Mean scores for each section are calculated: the higher the mean score of the sections, the better the usability and related acceptance of the app. The questionnaire was translated into German and validated [27]. For the study, the end-user version of the MARS (uMARS) was used, which has not yet been published but was already used in another study [22, 28]. For this study, the sections were evaluated separately and assigned based on the content of usability, feasibility or acceptance.

### SUS

The SUS is a questionnaire that asks about the subjective usability of a system. It consists of ten items, each of which can be rated on a five-point scale from 1 “strongly disagree” to 5 “strongly agree” [21]. SUS scores have a range of 0 to 100, values of 68 or more indicate a good usability [23].

### Number of reported trainings

As a proxy for acceptance and feasibility, the number of reported trainings was used. It has been assumed that from the individuals with axSpA report all completed training in the *Trainingslog*. In order to be able to compare the training entries in the *Trainingslog* with the training actually completed, the participants had to document the performed training in a paper-training diary (“gold standard”) as a weekly review. At the end of the test phase, screenshots of all entries in the *Trainingslog* were sent to the study authors and analysed with the entries in the paper weekly review.

### Online focus groups/interview

In order to deepen and specify the quantitative results, qualitative data collection was conducted following the questionnaires. The aim of the focus groups was to capture specific errors, gaps and needs of the *Trainingslog*. PTs and individuals with axSpA were invited via email to separate online semi-structured focus groups, which were prepared based on the initial findings of the quantitative data exploring usability, feasibility and acceptance. The researcher explained the aim of the study and presented a summary of the quantitative data. Subsequently, the participants were invited to discuss and interpret the findings. Further, deepening questions were asked to get the full picture of the participants’ perceptions and opinions. The focus groups were conducted online via MS Teams [29] in Swiss German and were audio recorded. Technical support was available before and during the focus groups meeting. For those who could not participate in the focus groups, an online interview was offered. The focus groups and interview were conducted by NP (PT), previously unknown to the participants.

### Data analysis

The quantitative data was analysed using descriptive statistics using RStudio (version: 2023.03.0 + 386). Usability was defined at over 50/100 points in the SUS for marginal user-friendliness [30]. For the uMARS, a mean of ≥ 3 points was considered acceptable [26]. Furthermore, the frequency of the use of the *Trainingslog* was assessed by the discrepancy between completed training written on paper and actual entries in the *Trainingslog*. Here, an agreement of 70% was expected. Data was presented as mean and standard deviations, medians, interquartile ranges, counts and percentages as appropriate. Boxplots were used to summarize scores of the uMARS.

For the qualitative data analysis, MAXQDA software was used. The focus group meetings and interviews were transcribed and proofread by the researcher NP. Subsequently, data was analysed using the structured thematic qualitative content analysis, as described by Kuckartz [30]. Thematic main categories were created deductively based on the research objectives and sub-categories were developed inductively [30]. Coding was done by one person from the research team, 20% of the transcripts were additionally analysed by a second person to ensure consistency and significance of codes. Further, data were reflexively discussed within the group including peer-debriefing in order to increase the credibility of findings. Finally, thematic summaries were described for the main aspects acceptance, feasibility and usability.

## Results

### Participants

A total of eleven PTs and 15 individuals with axSpA were included in the study. During the test phase, four participants left the study due to the high time expenditure and one individual with axSpA was excluded from the study due to the lack of testing the *Trainingslog*. One PT was not a SVMB-group leader but was experienced in treating individuals with axSpA. All PTs and individuals with axSpA completed both the SUS and uMARS questionnaires. Seven (70%) individuals with axSpA completed the paper-based training diary. Eight PTs (88%) and nine individuals with axSpA (90%) took part in the online focus groups (four PTs, six individuals with axSpA) or online interviews (four PTs, three individuals with axSpA of which one was face-to-face by request of the intervieew). Except one PT, all participants exhibited a high proficiency related to technology based on their MDPQ-16 scores. Characteristics for the study participants are shown in Table 1.

**Table 1:** Participants characteristics

Characteristics	individuals with axSpA	PTs
n	10	11
Gender, female, n (%)	6 (60)	9 (82)
Age, years [SD]	48 [13.4]	52.5 [15.6]

axSpA = axial Spondyloarthritis, PT = physiotherapists, n = number, SD = standard deviation

### Quantitative results

#### Usability

**Fig. 1:**
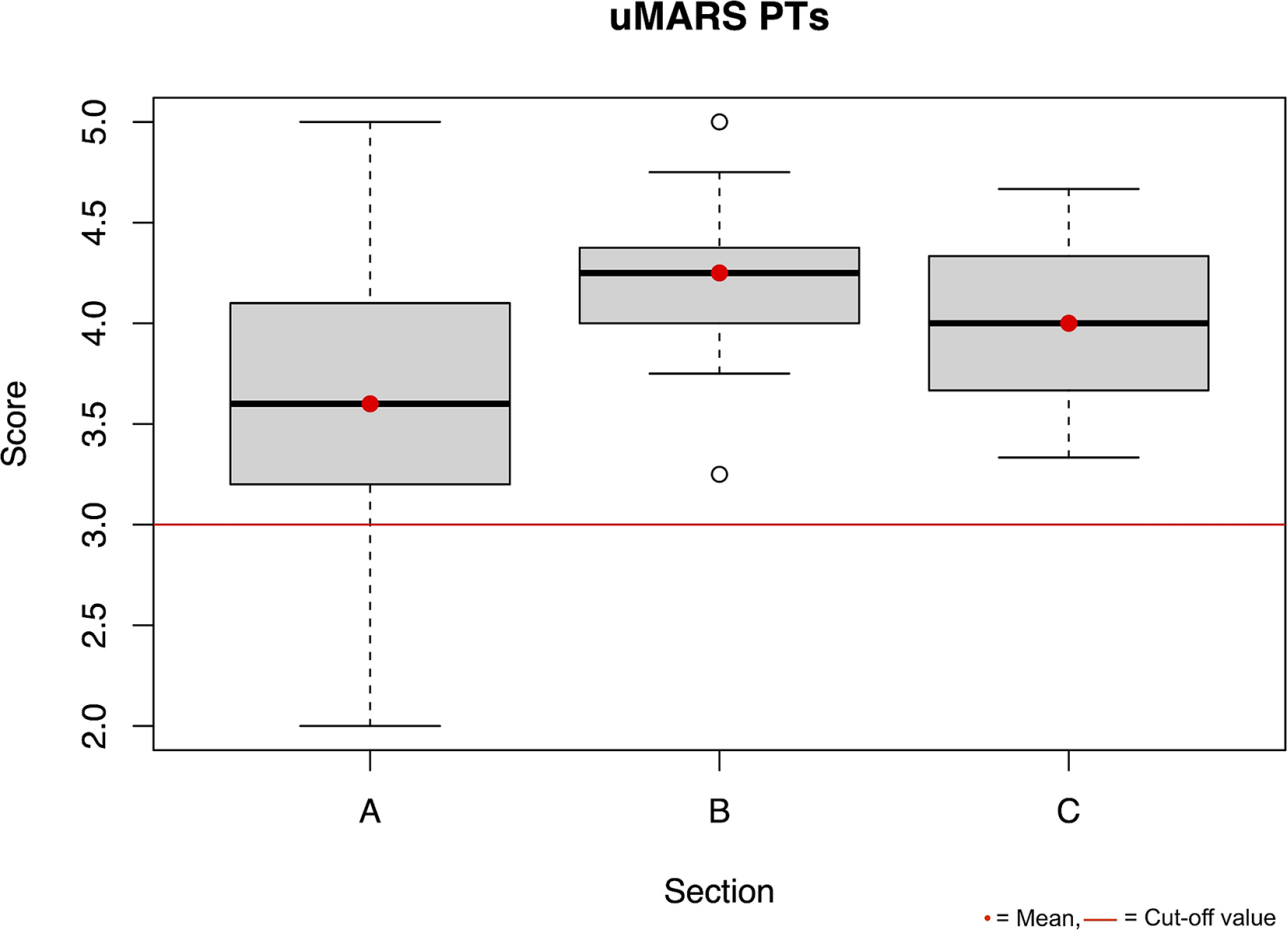
Boxplot uMARS PTs Section A engagement, B functionality, C aesthetics

**Fig. 2:**
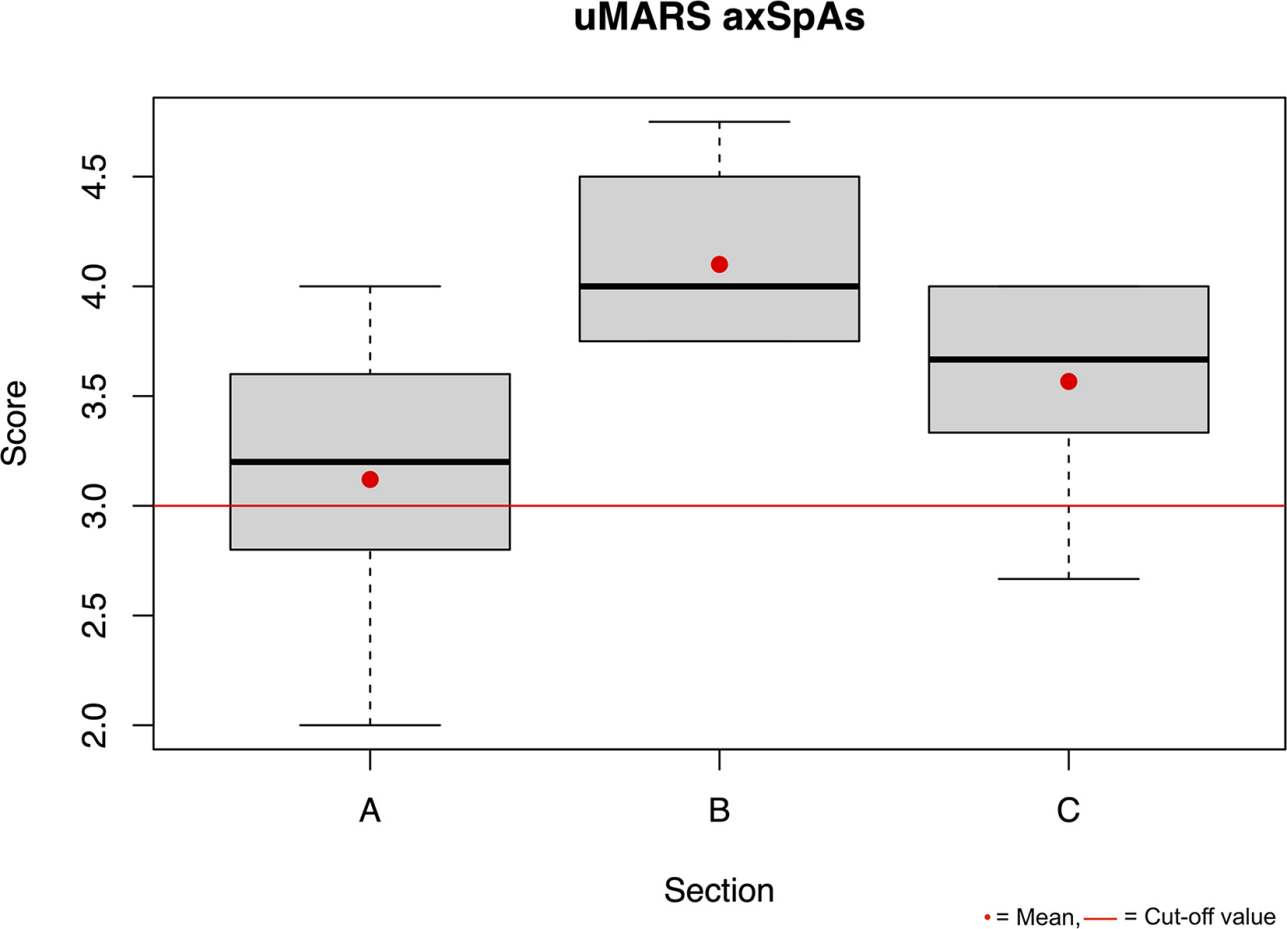
Boxplot uMARS axSpAs Section A engagement, B functionality, C aesthetics

The usability of the *Trainingslog* was rated with a mean SUS score of 82.50 (SD 21.76) by the PTs and with a mean SUS score of 77.00 (SD 9.34) by the individuals with axSpA. Furthermore, in the graphical display (Figs. 1 and 2) it can be seen that the mean value of the uMARS in all sectors is above 3 points.

#### Feasibility

The mean value of the uMARS section B was 4.2 (SD 0.49), rated by PTs and 4.1 (SD 0.38), rated by the individuals with axSpA.

#### Acceptance

**Fig. 3:**
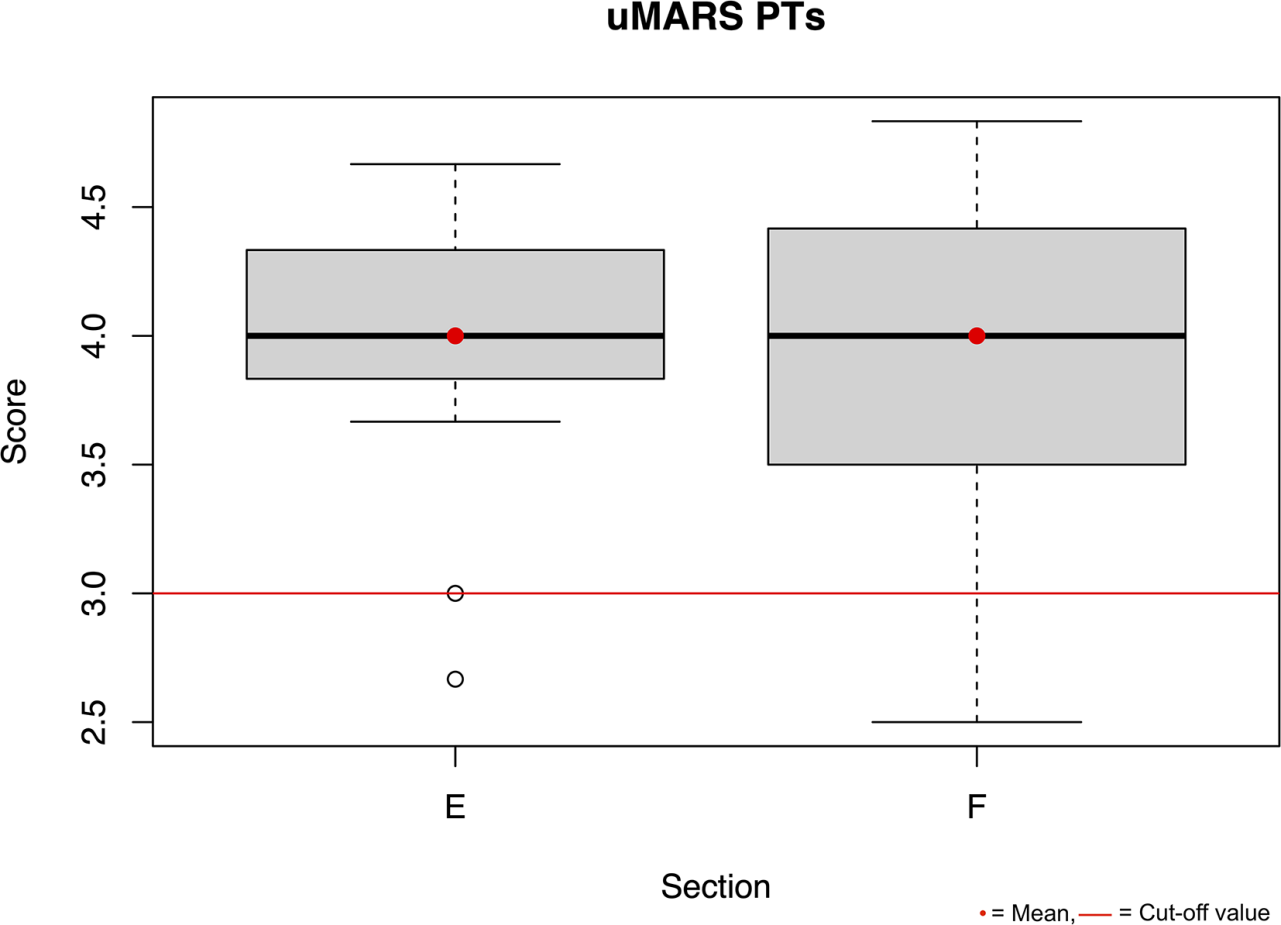
Boxplot uMARS PTs Section E app subjective, F app specific

**Fig. 4:**
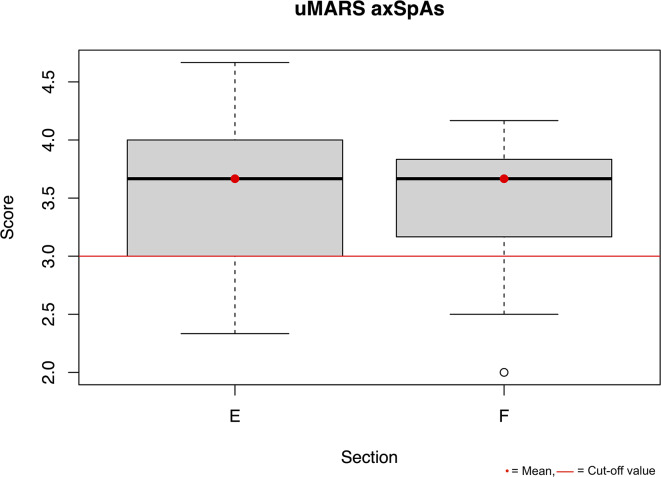
Boxplot uMARS axSpAs Section E app subjective, F app specific

Measuring acceptance of the *Trainingslog* a mean value of over 3 in Sector E and F of the uMARS was achieved for both groups. The results are presented in the boxplot Figs. 3 and 4. The comparison of the training entries between the paper-training diary and the *Trainingslog* has a match rate of 59.86% in the group of individuals with axSpA.

### Qualitative results

In general, the *Trainingslog* was described as useful and easy to use. However, the web-based link form of the *Trainingslog* was perceived as a main barrier, which is mainly due to the time-consuming navigation path to the *Trainingslog*. In addition, the *Trainingslog* was easily forgotten due to the lack of visibility on the smartphone. The app form was clearly favored by PTs and individuals with axSpA.

The *Trainingslog* was tested on tablets, computers and smartphones. It worked on all devices without any reported technical errors.

The PTs considered using the *Trainingslog* with ‘willing’ patients in the future. In the group of individuals with axSpA, three (33%) out of nine would not use it in the future and six (66%) out of nine individuals would only use it in the app-based form. The *Trainingslog* was seen by the PTs and individuals with axSpA as a motivational tool for more exercise.

The qualitative results are summarized in Table 2 based on the deductively created codes. A list of minor adaptation requests resulting from the focus groups can be found in the appendix.

**Table 2:** Qualitative results

Usability	PTs	Individuals with axSpA
In general	– Positive: • Useful and easy to use • Simple • Positive impression • Link to Rheumafit (website providing various exercises) – Desired: App form	– Positive: • Useful and easy to use • Simple • No unnecessary functions • Satisfied with the general design – Desired: App form
Training entry function	– Positive: • Clear and useful • Appropriate and relatively complete training dimensions and selection of sports – Challenges: changes with the time specification, editing training entry, changes in intensity scale	– Positive: Easy to handle – Challenges: Difficult to assign the training to the correct training dimension, Changes with the time specification – Desired: More examples for certain trainings, additional sports can be entered manually and some sports can be saved as favourites, free text field for comments
Overview function	– Positive: • Easy to understand and clear • Motivating – Desired: Increased visibility and improve access, longer period of time for the overview, percent target bar that can be achieved by reaching over 100%	– Positive: • For majority easy to understand and clear – Challenges: • Display of the evaluation was a bit too small • Unclear how and when they would reach the weekly target – Desired: Overview need to be larger and include several days
Information function	– Challenges: Not found by majority – Desired: • Contact address for questions • Short explanations for better usability	– Challenges: Not found by some test people – No need for more information or contact
Login and registration	– Worked well	
Request addition functions	– Link to a smartwatch or another app – Reminder function in the form of a push notification – Reward system – Questionnaire on mobility or pain – Integrated timer	– Link to a smartwatch or another app – Reminder function in the form of a push notification – Reward system – Possibility of exchanging information or networking with other individuals with axSpA – Able to enter individual training goals – Integrated exercise suggestions in the Trainingslog

Feasibility	PTs	axSpA
In general	– Navigation and functions worked well – Feasible for patients	– Navigation and functions worked well – Worked on all terminals
Devices	– Mainly smartphone	– Tablets, laptops/ computer, smartphone
Everyday life	– Time required for trainings entry is feasible – Assigning a sport according to the correct training dimension is seen as a possible challenge for patients	– Time consuming for training entry from 1 to 10 minutes – Easy to integrate into everyday life

Acceptance	PTs	axSpA
In general	– Motivating tool	– Motivating tool – Positive influence on exercise behaviour
Future Use	– Usable in future for “willing” people – Usable for other patient groups	– Daily or sporadic use – 3 people would not use in future – 6 out of 9 would only use the Trainingslog in app based form
Barriers	– Trainingslog as web based link	– Trainingslog as web based link
Wish	– Trainingslog in app based form	– Trainingslog in app based form

PT = physiotherapists, axSpA = axial Spondyloarthritis

## Discussion

To the best of our knowledge the *Trainingslog* is the first digital training diary for individuals with axSpA to be evaluated for usability, feasibility and acceptability. Overall, the *Trainingslog* has a good usability and feasibility among individuals with axSpA and PTs. However, the acceptance is strongly dependent on the form (web-based link or app) of the *Trainingslog*.

### Usability

The quantitative data of this study show that the usability of the *Trainingslog* is acceptable. According to Bangor et al. interpretation scale of the SUS, the *Trainingslog* achieves good usability for both groups [31]. The usability could also be further confirmed by the analysis of the qualitative data. In particular, the simple design as well as easy handling of the *Trainingslog* was appreciated by both groups. Both groups criticised that the *Trainingslog* is provided as a web-based link form and suggested it to be provided in app form. In a previous study conducted by Rausch et al., participants primarily desired a digitally-based training diary [11]. However, the clear preference for an app version was then still unknown. Both groups suggested supplementary functions such as push notifications, a reward system, or compatibility with a smartwatch. The importance of reminder functions and gamification elements such as praise and rewards to increase adherence to web-based information were also mentioned in the systematic review by Kelders et al., which evaluated the adherence to web-based chronic disease interventions [32].

### Feasibility

The *Trainingslog* was rated as feasible, with both groups achieving a mean score above three points in the uMARS. The focus groups confirmed that all functions of the *Trainingslog* were feasible, and no error messages were reported. In addition, according to the focus groups’ findings, the *Trainingslog* can be accessed via a variety of devices, including computers, tablets and smartphones. The group of individuals with axSpA reported that the *Trainingslog* is easy to integrate into their everyday life.

### Acceptance

With a mean value of over three in the uMARS, the acceptance of the *Trainingslog* was acceptable in both groups. It is striking that none of the subjects in the group of individuals with axSpA would buy the *Trainingslog* if they had to be paid for, and only 9% of the PTs group would buy the *Trainingslog*. However, this result is negligible, as the *Trainingslog* would be available free of charge to the end users and members of SVMB. Participants reported 60% of their training-entries in the paper-based diary also in the *Trainingslog*. The set target was that training sessions would have been reported in both diaries, which did not work. This may have two possible reasons: the web-based solution did to satisfy the users, or the two proxies of the real training frequency are not as comparable as previously thought. However, participants received a reminder only for the paper-training diary by weekly e-mails.

The qualitative results show that the *Trainingslog* is mainly seen as a motivating factor for exercise. Some of the individuals with axSpA reported that the *Trainingslog* has positively influenced their exercise behaviour during the test phase. The positive influence of self-monitoring on physical activity behaviour has already been proven by other studies [13].

The acceptance of the *Trainingslog* in both groups depended heavily on the form of the *Trainingslog*. It was clearly mentioned in both groups that the *Trainingslog* in app form is prioritised and if it will be used in the future depends on it. The primary barrier posed by a web-based link was the lack of visibility on smartphones and the time-consuming navigation required to access the link. This temporal barrier is consistent with the findings of Peng et al., who identified time and effort as the primary barriers to the continued use of apps [33]. The argument of visibility on the smartphone facilitates regular registry of a training entry can be supported with the behavioural change technique “add objects to the environment” described by Michie et al. [34]. However, many participants in the study were able to envision using the *Trainingslog* in the future, especially in app form. Additionally, the group of PTs can also imagine using the *Trainingslog* in therapy to support patients in the future.

### Comparison with other mobile health technologies

The qualitative results obtained in this study have parallels with those reported in previous studies on mobile health technologies for chronic disease management. In contrast to the *Trainingslog*, many other mobile health technologies already exist in an app-based version. It was particularly noticeable that the push notification function is also desired or highly appreciated in other studies [35, 36]. In addition, other articles on mobile health apps for rheumatic diseases also mention additional features such as passive sensors that measure movement or gamification that potentially increase the adherence of an app [37, 38].

### Recommendations for further development of the *Trainingslog*

Based on the results, it is recommended to change the *Trainingslog* into an app form to increase user acceptance. Furthermore, it is recommended to implement the small adjustment requests from the list in the appendix.

### Strengths

A strength of the study was the close collaboration of the scientific and technical developers during the development and research process of the Trainingslog. The study included end-users of the *Trainingslog*, such as individuals with axSpA and PTs, which is relevant for the further use and development of a mobile health technology [39]. The required sample size for usability studies has been reached.

### Limitation

The study has several limitations that must be considered when interpreting the results. Firstly, acceptance was measured by comparing the training entries in the *Trainingslog* and the training entries on the paper weekly review. The result was very unclear and should be interpreted with caution because the cut-off was set by the author of the study and the reminder system was not the same for both methods. However, some days had more entries in the *Trainingslog* than in the weekly overview. This could be related to a recall bias and reporting bias. We used the paper-diary as “gold-standard”, however, we are aware that objective measures of physical activity (such as accelerometer) would have been more appropriate to capture the true number of exercise sessions.

Secondly, the measurement instruments of the quantitative results have some limitations. The German versions of the uMARS and SUS have not yet been validated, and while they were used in other studies, the validity of the results is uncertain and must be interpreted with caution. Further, the questions in uMARS were developed for an app, which may have led to confusion among some individual’s subjects.

Thirdly, transferring the results to the target population of individuals with axSpA and PTs is limited as the study sample does not represent all end-users. Only one person with a low proficiency related to technology was included in the study, which makes it difficult to transfer the findings to people with different levels of proficiency related to technology. Furthermore, three individuals dropped out during testing period due to lack of time (*n* = 3), which is also a common barrier for performing exercises [40]. In future, it has to be taken into account that persons who have no physically active lifestyle may need more support to feel confident to fulfil the instructions. One person was excluded because she had not tested the *Trainingslog* before participating in the focus group due to a misunderstanding of the instructions. In future, instructions and related goals have to be clearer.

Fourthly, more measures of trustworthiness, as described by Steinke [41], could have been taken, such as choosing quotes for ensuring empirical anchoring, returning transcripts and findings to participants for comments, or performing the full coding process by two researchers. However, the intersubjective comprehensibility was enhanced by stringent documentation of the process, and findings were regularly discussed within the group including experts for the research methodology, development of technology, and the individual with axSpA.

## Conclusion/potential

The findings of the study indicate that the *Trainingslog* has a good usability and feasibility among individuals with axSpA and PTs with high proficiency related to technology. The acceptance could be improved by providing *Trainingslog* as an app. The *Trainingslog* has the potential to be used for self-monitoring of physical activity by individuals with axSpA.

## Electronic supplementary material

Below is the link to the electronic supplementary material.

Supplementary Material 1

## Data Availability

The datasets used and/or analysed during the current study are available from the corresponding author on reasonable request.
