# Supplementary material for: Evaluation of usability, feasibility and acceptance of the digital training diary Trainingslog for individuals with axSpA: a mixed-method study
Source: BMC Rheumatol. 2025 Feb 18;9:18. doi: 10.1186/s41927-025-00463-5 (PMC11834626; doi:10.1186/s41927-025-00463-5)
Supplement: Supplementary file 1 — Supplementary Material 1 [file 41927_2025_463_MOESM1_ESM.docx]

## Recommendations for further development

| Preferred type of offering the *Trainingslog*:   - App form |
| --- |
| Design:   - Larger font - Endurance in a colour other than red, for example, pink - Stronger and larger colours points in the calendar |
| Function training entry:   - If no hour is entered, a zero should be entered automatically - Possibility to enter a time unit smaller than 5 minutes - Strength training can also be entered as minutes - Split a training session into different training dimensions (e.g. 20 min strength, 30 min endurance etc.) - Manually add additional sports - Save sports as favourites - Training entries should be editable - Reduce number of smiley's of the intensity scale - Be able to record additional sensations such as pain |
| Function Overview:   - More present / quicker to find - Percentage bar of strength should only be filled when all major body parts have been trained. - Percentage bar should be able to grow further than 100% - Additional 6-month overview - Show daily overview with more days - Display points on calendar view larger and with stronger colours |
| Function information / contact   - More conspicuous information button, better visible - Uniform click on information buttons - Short explanation of the information buttons when used for the first time |
| Additional functions   - Possibility of push notification / reminder function - Supplemented with a reward system (e.g. cups or stars) - Questionnaire on mobility or pain as a progress parameter - Possibility to connect with a smartwatch or activity tracker - Integrate a timer for training recording - Possibility to set individual training goals - Text field for additional comments (e.g. reason for not training: sick) - Integrate training suggestions into *Trainingslog* (link to Rheumafit already exists) - Integrating an exchange function with other individuals with axSpA (e.g. chat function) |

Interface *Trainingslog*

| 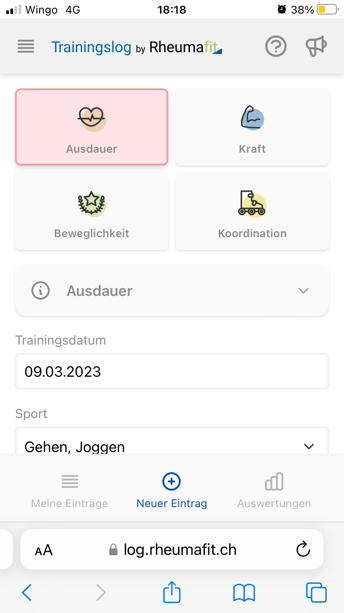 | 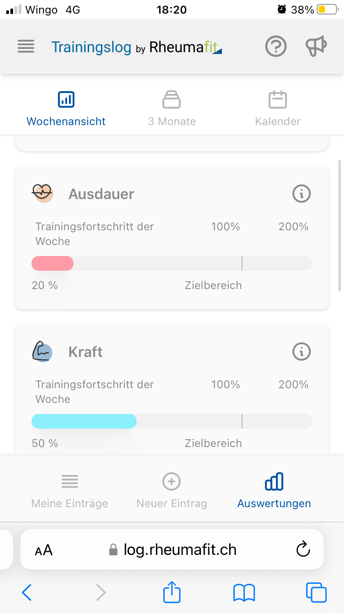 | 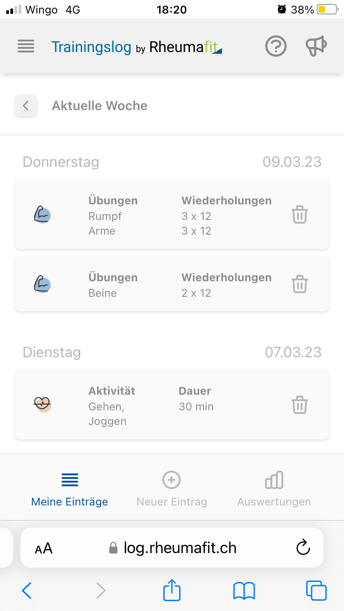 | 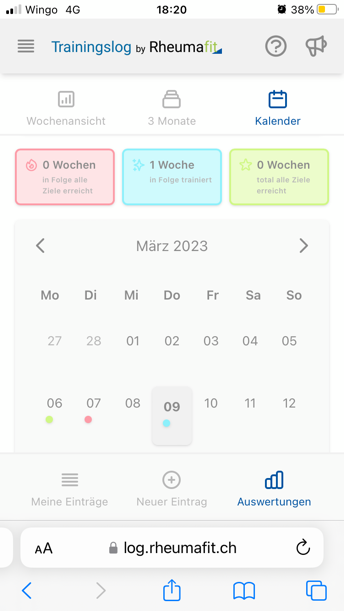 |
| --- | --- | --- | --- |
